# PERSPECTIVES ON POLICIES TO IMPROVE EQUITY IN HOSPITAL AT HOME

**DOI:** 10.1093/geroni/igae098.3879

**Published:** 2024-12-31

**Authors:** Jeannys Nnemnbeng, Abigail Baim-Lance, Brittni Howard, Julia Hart, Bruce Leff, David Levine, Albert Siu, Ksenia Gorbenko

**Affiliations:** Icahn School of Medicine at Mount Sinai, New York, New York, United States; Icahn School of Medicine at Mount Sinai, New York, New York, United States; University of Massachusetts at Amherst, Amherst, Massachusetts, United States; Icahn School of Medicine at Mount Sinai, New York, New York, United States; Johns Hopkins Medicine, Baltimore, Maryland, United States; Brigham and Women’s Hospital, Boston, Massachusetts, United States; Icahn School of Medicine at Mount Sinai, New York, New York, United States; Icahn School of Medicine at Mount Sinai, New York, New York, United States

## Abstract

Hospital at Home (HaH) delivers acute hospital-level care at home as a substitute for traditional hospital care. Compared to traditional hospital care, HaH care demonstrates better patient satisfaction and care experience, lower readmission rates, and lower costs of care. There has been limited research on equity issues in HaH care delivery. We explored HaH program leaders’ views on the policy landscape and opportunities for equitable delivery of HaH across the US. We conducted a qualitative study using semi-structured interviews with healthcare leaders (1-4 leaders per program) at 18 HaH programs diverse by geography (region, rurality), size, and safety-net status. We conducted interviews via Zoom and used rapid template analysis and focused coding to identify key themes. We identified four areas supporting equitable HaH care delivery: (1) Technology access to promote more complete broadband coverage, particularly in rural areas; (2) Medicaid expansion to allow the enrollment of Medicaid beneficiaries, a frequent recommendation to increase lower-income individuals’ access to the program, including younger age groups that did not typically participate and would benefit; (3) Workforce role diversity to align with the needs of communities and therefore to include services like paramedicine familiar with delivering care at home; and (4) Sustainable and broadening of HaH funding including making the waiver permanent and expanding support for a package to connect patients with community-based care at discharge. HaH success in advancing equitable care delivery requires greater policy support to ensure equal access and solid funding mechanisms to support its ongoing growth.

